# SoilGrids250m: Global gridded soil information based on machine learning

**DOI:** 10.1371/journal.pone.0169748

**Published:** 2017-02-16

**Authors:** Tomislav Hengl, Jorge Mendes de Jesus, Gerard B. M. Heuvelink, Maria Ruiperez Gonzalez, Milan Kilibarda, Aleksandar Blagotić, Wei Shangguan, Marvin N. Wright, Xiaoyuan Geng, Bernhard Bauer-Marschallinger, Mario Antonio Guevara, Rodrigo Vargas, Robert A. MacMillan, Niels H. Batjes, Johan G. B. Leenaars, Eloi Ribeiro, Ichsani Wheeler, Stephan Mantel, Bas Kempen

**Affiliations:** 1 ISRIC — World Soil Information, Wageningen, the Netherlands; 2 Faculty of Civil Engineering, University of Belgrade, Belgrade, Serbia; 3 GILab Ltd, Belgrade, Serbia; 4 School of Atmospheric Sciences, Sun Yat-sen University, Guangzhou, China; 5 Institut für Medizinische Biometrie und Statistik, Lübeck, Germany; 6 Agriculture and Agri-Food Canada, Ottawa (Ontario), Canada; 7 Department of Geodesy and Geoinformation, Vienna University of Technology, Vienna, Austria; 8 University of Delaware, Newark (DE), United States of America; 9 LandMapper Environmental Solutions Inc., Edmonton (Alberta), Canada; 10 Envirometrix Inc., Wageningen, the Netherlands; Pacific Northwest National Laboratory, UNITED STATES

## Abstract

This paper describes the technical development and accuracy assessment of the most recent and improved version of the SoilGrids system at 250m resolution (June 2016 update). SoilGrids provides global predictions for standard numeric soil properties (organic carbon, bulk density, Cation Exchange Capacity (CEC), pH, soil texture fractions and coarse fragments) at seven standard depths (0, 5, 15, 30, 60, 100 and 200 cm), in addition to predictions of depth to bedrock and distribution of soil classes based on the World Reference Base (WRB) and USDA classification systems (ca. 280 raster layers in total). Predictions were based on ca. 150,000 soil profiles used for training and a stack of 158 remote sensing-based soil covariates (primarily derived from MODIS land products, SRTM DEM derivatives, climatic images and global landform and lithology maps), which were used to fit an ensemble of machine learning methods—random forest and gradient boosting and/or multinomial logistic regression—as implemented in the R packages ranger, xgboost, nnet and caret. The results of 10–fold cross-validation show that the ensemble models explain between 56% (coarse fragments) and 83% (pH) of variation with an overall average of 61%. Improvements in the relative accuracy considering the amount of variation explained, in comparison to the previous version of SoilGrids at 1 km spatial resolution, range from 60 to 230%. Improvements can be attributed to: (1) the use of machine learning instead of linear regression, (2) to considerable investments in preparing finer resolution covariate layers and (3) to insertion of additional soil profiles. Further development of SoilGrids could include refinement of methods to incorporate input uncertainties and derivation of posterior probability distributions (per pixel), and further automation of spatial modeling so that soil maps can be generated for potentially hundreds of soil variables. Another area of future research is the development of methods for multiscale merging of SoilGrids predictions with local and/or national gridded soil products (e.g. up to 50 m spatial resolution) so that increasingly more accurate, complete and consistent global soil information can be produced. SoilGrids are available under the Open Data Base License.

## Introduction

There is a growing demand for detailed soil information, especially for global estimation of soil organic carbon [1–3] and for modeling agricultural productivity [4, 5]. Spatial information about soil water parameters is likely to become increasingly critical in areas affected by climate change [6]. Soils and soil information are also particularly relevant for the Sustainable Development goal target 15.3 of achieving Land Degradation Neutrality (LDN), as specified by the United Nations Convention to Combat Desertification (UNCCD; http://www.unccd.int), and are one of the main areas of interest of the FAO’s Global Soil Partnership initiative [7]. Folberth et al. [8] have recently discovered that accurate soil information might be the key to predicting either buffering or amplifying impacts of climate change on food production.

To reduce the gap between soil data demand and availability, ISRIC (International Soil Reference Information Centre)—World Soil Information released a Global Soil Information system called *“SoilGrids”*. The first version of SoilGrids (predictions at 1 km spatial resolution released in 2014), was, at the time, a ‘proof of concept’ demonstrating that global compilations of soil profiles can be used in an automated framework to produce complete and consistent spatial predictions of soil properties and classes [9]. Since the launch of the system in 2014, several colleagues have recognized and reported some of the limitations of the first version of the system. Mulder et al. [10] observed, using more detailed soil profile data and maps, that SoilGrids likely overestimated all low values for organic carbon content in France. Likewise, Griffiths et al. [11] reported underestimation of the pH in comparison to UK national data. The overestimation of low values happened mainly as an effect of limited fitting success (so that both high and low values are smoothed out). In addition, many of the artifacts visible in the Harmonized World Soil Database (HWSD) [12], which was used as one of the covariates to produce the first version of SoilGrids, e.g. country borders, were propagated to SoilGrids1km. Some users have also expressed concerns that the first version of SoilGrids did not provide predictions for arid and desert areas and hence can be considered an incomplete product [13].

To address these criticisms and concerns, we have re-designed and re-implemented SoilGrids with a particular emphasis on addressing methodological limitations of SoilGrids1km. Hence, our main objective was to build a more robust system with improved output data quality; especially considering spatial detail and attribute accuracy of spatial predictions. We implemented the following six key improvements:

1. We replaced linear models with tree-based, non-linear machine learning models to account for non-linear relationships—especially for modeling soil property–depth relationships—but also to be able to better represent local soil–covariate relationships. Predictions are now primarily data-driven. Much less time is spent on choosing models, which also reduces the complexity of producing updates.
2. We replaced single prediction models with an ensemble framework i.e. we use at least two methods for each soil variable to reduce overshooting effects.
3. We extended the initial list of covariates to include a wider diversity of MODIS land products and to better represent factors of soil formation. The spatial resolution of covariates was increased from 1 km to 250 m with the expectation that finer resolution will help increase the prediction accuracy.
4. We re-implemented the global soil mask using state-of-the-art land cover products [14]. The current soil mask now includes all previously excluded dryland and sand dune areas so that most of the land mask (> 95%) is represented.
5. The global compilation of soil profiles and samples used for model training was also extended. We added extra points for the Russian Federation, Brazil, Mexico and the Arctic circle; and re-visited data harmonization issues.
6. We created and inserted expert-based pseudo-points for a selection of parameters to minimize extrapolation effects in undersampled geographic areas lacking field observations, such as deserts, semi-deserts, glaciers and permafrost areas.

We present here the technical development and accuracy assessment of the updated SoilGrids system at 250 m resolution. In the following sections we describe the workflows used to generate spatial predictions and report results of model fitting and accuracy assessment based on 10–fold cross-validation. We conclude the article by suggesting some possible applications of this new data set and identifying possible future improvements. SoilGrids250m map layers are available for download via www.SoilGrids.org under the Open Database License (ODbL). GeoTiffs can also be obtained from ftp://ftp.soilgrids.org/data/.

## Methods and materials

### Target variables

SoilGrids provides predictions for the following list of standard soil properties and classes [9]:

- Soil organic carbon content in ‰ (g kg^−1^),
- Soil pH in H_2_O and KCl solution,
- Sand, silt and clay (weight %),
- Bulk density (kg m^−3^) of the fine earth fraction (< 2 mm),
- Cation-exchange capacity (cmol + /kg) of the fine earth fraction,
- Coarse fragments (volumetric %),
- Depth to bedrock (cm) and occurrence of R horizon,
- World Reference Base (WRB) class—at present, we map 118 unique soil classes, e.g. Plinthic Acrisols, Albic Arenosols, Haplic Cambisols (Chromic), Calcic Gleysols and similar [15]. This is about four times as many classes as in the previous version of SoilGrids,
- United States Department of Agriculture (USDA) Soil Taxonomy suborders—i.e. 67 soil classes [16].

**Fig 1:**
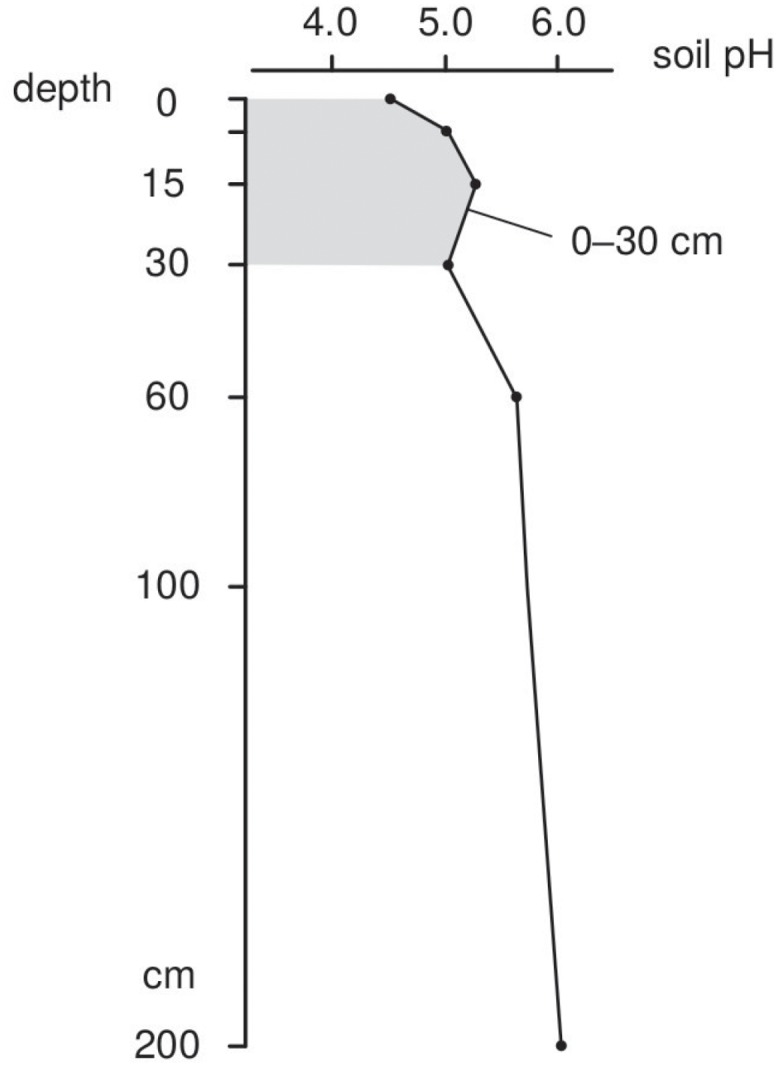
Standard soil depths following the GlobalSoilMap.net specifications and example of numerical integration following the trapezoidal rule.

We generated predictions at seven standard depths for all numeric soil properties (except for depth to bedrock and soil organic carbon stock): 0 cm, 5 cm, 15 cm, 30 cm, 60 cm, 100 cm and 200 cm, following the vertical discretisation as specified in the GlobalSoilMap specifications [17]. Averages over (standard) depth intervals, e.g. 0–5 cm or 0–30 cm, can be derived by taking a weighted average of the predictions within the depth interval using numerical integration, such as the trapezoidal rule:

$$\frac{1}{b-a}\int_{a}^{b}f\left(x\right)\;dx\approx \frac{1}{\left(b-a\right)}\frac{1}{2}\sum_{k=1}^{N-1}\left(x_{k+1}-x_{k}\right)\left(f\left(x_{k}\right)+f\left(x_{k+1}\right)\right)$$ (1)

where *N* is the number of depths, *x*_*k*_ is the *k*-th depth and *f*(*x*_*k*_) is the value of the target variable (i.e., soil property) at depth *x*_*k*_. For example, for the 0–30 cm depth interval, with soil pH values at the first four standard depths equal to 4.5, 5.0, 5.3 and 5.0, the pH is estimated as $\frac{1}{30\cdot 2}\cdot [(5-0)\cdot (4.5+5.0)+(15-5)\cdot (5.0+5.3)+(30-15)\cdot (5.3+5.0)]/30\cdot 0.5=5.083$ (Fig 1).

**Fig 2:**
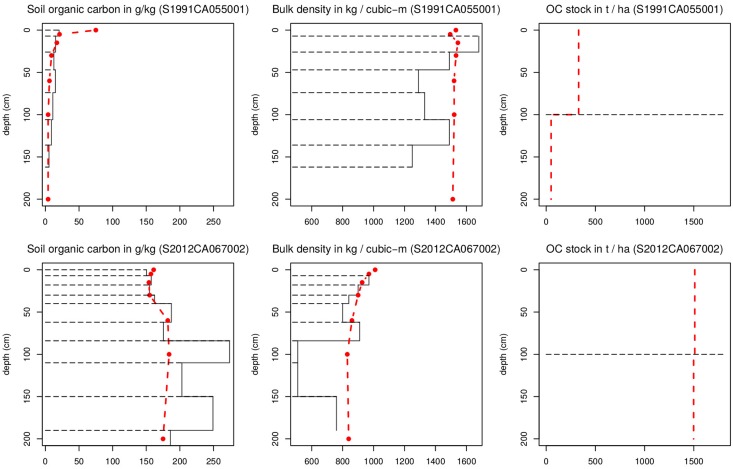
Example of soil variable-depth curves: Original sampled soil profiles (black rectangles) vs predicted SoilGrids values at seven standard depths (broken red line), and predicted soil organic carbon stock for depth intervals 0–100 and 100–200 cm. Locations of points from the USDA National Cooperative Soil Survey Soil Characterization database: mineral soil S1991CA055001 (-122.37°W, 38.25°N), and an organic soil profile S2012CA067002 (-121.62°W, 38.13°N).

Based on predictions of soil organic carbon content, bulk density, and coarse fragments, we also derived soil organic carbon stock (tha^−1^) for the six GlobalSoilMap standard depth intervals following the standard approach [9, 18]. Fig 2 shows an example of observed vs predicted values and corresponding derived soil organic carbon stock for 0–1 m and 1–2 m depths.

Model fitting and spatial prediction of depth to bedrock is based also on water well drilling data. Model fitting and spatial prediction of soil depth to bedrock variables is explained in detail in Shangguan et al. [19].

We set the reference soil surface at the air/soil boundary, as per FAO [20], hence all soil material is included. Some national soil survey teams (and also earlier versions of the FAO standards) define 0 cm depth at the start of the mineral soil, i.e. just below the O or the P (peat) horizon. Consider for example the following sample soil profile from Canada [21]:

hor  top  bottom  bd  orgcarb

LFH   -12  0     0.07  48.1

Ae    0    11      1.3    0.6

AB   11  25      1.53  0.4

Bt     25  44      1.62  0.4

which shows that the vertical coordinates of the organic layer of this soil site are negative (LFH indicates Litter—Fermentation—Humus); orgcarb indicates soil organic carbon, bd is the bulk density and top and bottom are the upper and lower horizon depth in cm). Therefore, to avoid vertical mismatches between different national systems, all systems that put the zero level at the start of the mineral soil have been adjusted to a reference with the zero level at the air/soil boundary. For the example soil profile from Canada this means that 12 cm was added to all top and bottom values (in the example above, there is a significant discontinuity in values in organic carbon that drops from 48.1% to 0.6% within 12 cm of depth).

### Input profile data

**Fig 3:**
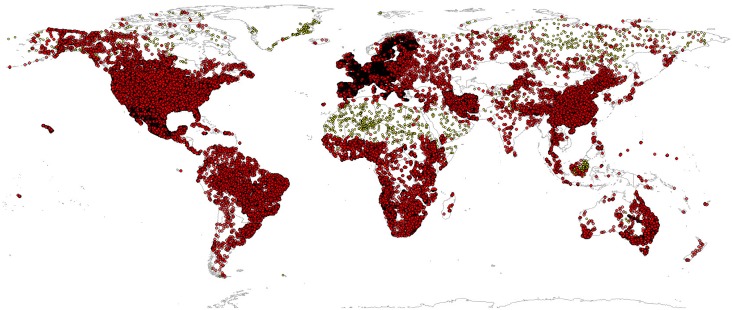
Input profile data: World distribution of soil profiles used for model fitting (about 150,000 points shown on the map; see acknowledgments for a complete list of data sets used). Yellow points indicate pseudo-observations. For the majority of points shown on this map, laboratory data can be accessed from ISRIC’s World Soil Information Service (WoSIS) at http://wfs.isric.org/geoserver/wosis/wfs.

For model building, we used soil profile data from ca. 150,000 unique sites spread over all continents (Fig 3; see acknowledgments for a full list). These have been imported, cleaned and merged into a single global compilation of soil points with unique column names and IDs.

Preparation of the global compilation of standardized soil training points took several months of work. The translation and cleaning up of soil properties and soil classes took a large amount of time. About 15–20% of the original soil profile data was only reported using a national classification system, e.g. the Canadian and Brazilian classification systems. Since some information is better than none, where possible we translated national classification systems to the two international (World Reference Base and USDA) classification systems. For translation we used published correlation tables either reported in Krasilnikov et al. [22] or reported on the agency websites; see e.g. correlation of Canadian Soil Taxonomy published (http://sis.agr.gc.ca/cansis/taxa/) and correlation of the Brazilian classification system (http://www.pedologiafacil.com.br/classificacao.php). We also consulted numerous local soil classification experts and requested their feedback and corrections in the (online) correlation tables (distributed via Google spreadsheets). Some national classification systems, such as the Australian soil classification system, are simply too different from the USDA and WRB systems to allow satisfactory correlation. These data were therefore not used. The full list of correlation tables is available from ISRIC’s github account at https://github.com/ISRICWorldSoil.

Another time-consuming operation was merging laboratory measurements and field observations and their harmonization to a standard format. In some cases missing values in the original tables had been coded as "0" values, which can have a serious influence on prediction models; in other cases we implemented and applied functions to locate and correct typos and other gross errors. Some variables, such as soil organic carbon, needed to be converted either from soil organic matter (e.g. divide by 1.724) and/or by removing CaCO_3_ (Calcium carbonates) from total carbon. Nevertheless, the majority of soil variables from various national soil profile data bases appeared to be compatible and relatively easy to merge—soil scientists across continents do measure similar things, but often express the results using different measurement units, vocabularies and standards.

We imported all original tables *as-is*, next documented all conversion functions through R scripts (available via ISRIC’s github account), to accommodate reproducible research and facilitate that conversion functions may, in the future, be further modified and improved. The majority of the points (excluding LUCAS points and other data sets with specific restricting terms of use) and legends used for model building and for producing SoilGrids are also available for public use via ISRIC’s WoSIS Web Feature Service (http://www.isric.org/data/wosis) and/or the ISRIC’s institutional github account.

### Expert-based pseudo-observations

Even though the input training point data are extensive and cover most continents and climatic zones, some large areas that have extreme climatic conditions and/or have very restricted access, are significantly undersampled. This occurs largely in the following five types of areas:

1. Semi-arid and arid lands, deserts and sand dunes,
2. Mountain tops, steep slopes of mountains and similar inaccessible areas,
3. Areas covered by ice and/or snow, i.e. glaciers,
4. Inaccessible tropical forest,
5. Areas governed by totalitarian and hostile regimes, with military conflicts or war.

It might seem obvious to soil surveyors that there is no soil organic carbon in the top 2 m of the active sand dunes of the Sahara, but any model fitted without observations in the Sahara could result in dubious extrapolation and questionable predictions. In addition, relationships across transitional areas—from semi-arid zones to deserts—can be difficult to represent without enough points at both edges of the feature space. Some sand dunes in the USA have been actually sampled and analyzed in the laboratory. For example, Lei [23] has shown that sand dunes in the Mojave desert have an average pH of 8.1, 98% sand and 0% organic carbon. Again, although it might seem obvious that deserts consist mainly of sand, and that steep slopes without vegetation are either very shallow or show bedrock at the surface, the model is not aware of such expert knowledge and hence such features need to be ‘numerically represented’ in the calibration dataset. We therefore decided, instead of masking out all such areas from soil mapping, to insert pseudo-observations and fill gaps in the feature space for the first four of the five types of areas listed above, i.e. to add pseudo-observations to the training dataset, which we then use for model building.

We used the following data sources to delineate sand dunes, bare rock and glaciers and produce their respective land masks:

- Sand dunes mask—To delineate the global distribution of sand dunes we used mean annual long-term surface temperature generated from the MODIS LST data product (MOD11A2), long-term MODIS Mid-Infrared (MIR) band (MCD43A4) and a slope map. After visual inspection of the border of the Sahara desert, it was clear that sand dunes can be relatively accurately delineated using MIR reflectance, mean daily annual temperature (> 25°C) and a slope map (< 25 rad).
- Bare rock mask—To delineate bare rock we also used the MODIS MIR band (MCD43A4) and a slope map. Bare rock or dominantly rocky areas show high MIR surface reflectance and are associated with steep slopes (> 32 rad). To the initial mask map estimated using MODIS MIR band and slope map, we also added bare rock areas from more detailed maps available for some countries, such as Iceland and northern Europe [19].
- Glaciers mask—To represent global distribution of glaciers we used the GLIMS Geospatial Glacier Database [24].

For each of the three masks we then generated randomly 100–400 points based on the relative global extent and assigned soil properties and soil classes accordingly (e.g. in the case of WRB’s Protic Arenosols for sand dunes, Lithic and Rendzic Leptosols for bare rock areas, Cryosols for areas adjacent to glaciers; in the case of USDA’s Psamments for sand dunes, Orthents for bare rock areas and Turbels for glaciers; for sand dunes we also inserted estimated values of 0 soil organic carbon, 98% sand and 0% coarse fragments). For model training for predicting soil classes we also used pseudo-observations generated from the best available soil polygon maps: for poorly accessible tropical forest areas, such as Indonesia, we used the Land information system of Kalimantan [25], and for northern latitudes, i.e. to represent permafrost soils, the Northern Circumpolar Soil Carbon Database was used [26].

When inserting pseudo-observations we tried to follow three simple rules of thumb to minimize any negative effects:

- keep the relative percentage of pseudo-points small i.e. try not to exceed 1–2% of the total number of training points,
- only insert pseudo-points for which the actual ground value is known with high confidence, e.g. sand content in sand dune areas,
- if polygon maps are used to insert pseudo-observations, we tried to use the most detailed soil polygon maps and focus on polygons with very high thematic purity.

### Soil covariates

**Fig 4:**
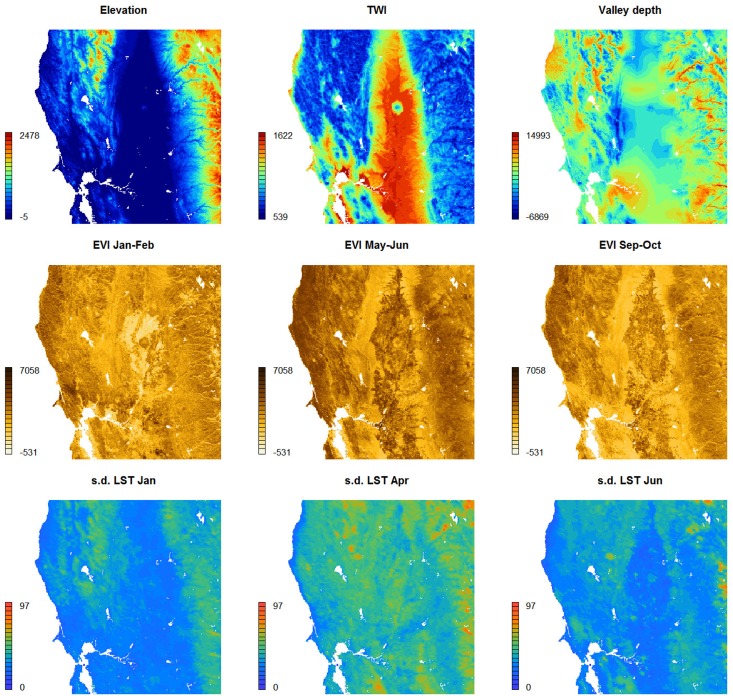
Examples of covariates used to generate SoilGrids: TWI is the Topographic Wetness Index (values multiplied by 100), EVI is the MODIS Enhanced Vegetation Index (values multiplied by 10,000), s.d. LST is the long-term standard deviation of MODIS Land Surface Temperatures (values in Celsius degrees). Location: San Francisco bay area, California. Size of the bounding box is 300 by 300 km.

As covariate layers for producing SoilGrids250m predictions we used an extensive stack of covariates, which are primarily based on remote sensing data. These include (see e.g. Fig 4):

- DEM-derived surfaces—slope, profile curvature, Multiresolution Index of Valley Bottom Flatness (VBF), deviation from Mean Value, valley depth, negative and positive Topographic Openness and SAGA Wetness Index—all based on the global merge of SRTMGL3 DEM and GMTED2010 [27]. All DEM derivatives were computed using SAGA GIS [28],
- Long-term averaged monthly mean and standard deviation of the MODIS Enhanced Vegetation Index (EVI). Derived using a stack of MOD13Q1 EVI images [29],
- Long-term averaged mean monthly surface reflectances for MODIS bands 4 (NIR) and 7 (MIR). Derived using a stack of MCD43A4 images [30],
- Long-term averaged monthly mean and standard deviation of the MODIS land surface temperature (daytime and nighttime). Derived using a stack of MOD11A2 LST images [31],
- Long-term averaged mean monthly hours under snow cover based on a stack of MOD10A2 8-day snow occurrence images [32],
- Land cover classes (cultivated land, forests, grasslands, shrublands, wetlands, tundra, artificial surfaces and bareland cover) for the year 2010 based on the GlobCover30 product by the National Geomatics Center of China [14]. Upscaled to 250 m resolution and expressed in percent of pixel coverage,
- Monthly precipitation images derived as the weighted average between the WorldClim monthly precipitation [33] and GPCP Version 2.2 [34],
- Long-term averaged mean monthly hours under snow cover. Derived using a stack of MOD10A2 8-day snow occurrence images,
- Lithologic units (acid plutonics, acid volcanic, basic plutonics, basic volcanics, carbonate sedimentary rocks, evaporite, ice and glaciers, intermediate plutonics, intermediate volcanics, metamorphics, mixed sedimentary rocks, pyroclastics, siliciclastic sedimentary rocks, unconsolidated sediment) based on Global Lithological Map GLiM [35],
- Landform classes (breaks/foothills, flat plains, high mountains/deep canyons, hills, low hills, low mountains, smooth plains) based on the USGS’s Map of Global Ecological Land Units [36].
- Global Water Table Depth in meters; after Fan et al. [37],
- Long-term averaged mean monthly MODIS Flood Water based on the NRT Global MODIS Flood Mapping Flood Water product (http://oas.gsfc.nasa.gov/floodmap/),
- Landsat-based estimated distribution of Mangroves; after Giri et al. [38],
- Average soil and sedimentary-deposit thickness in meters; after Pelletier et al. [39].

These covariates were selected to represent factors of soil formation according to Jenny [40]: climate, relief, living organisms, water dynamics and parent material. Out of the five main factors, water dynamics and living organisms (especially vegetation dynamics) are not trivial to represent as these operate over long periods of time and often exhibit chaotic behaviour. Using reflectance bands such as the mid-infrared MODIS bands from a single day, would have little use to soil mapping for areas with dynamic vegetation, i.e. with strong seasonal changes in vegetation cover. To account for seasonal fluctuation and for inter-annual variations in surface reflectance, we instead used long-term temporal signatures of the soil surface derived as monthly averages from long-term MODIS imagery (15 years of data). We assume here that, for each location in the world, long-term average seasonal signatures of surface reflectance or vegetation index provide a better indication of soil characteristics than only a single snapshot of surface reflectance. Computing temporal signatures of the land surface requires a considerable investment of time (comparable to the generation of climatic images vs temporary weather maps), but it is possibly the only way to represent the cumulative influence of living organisms on soil formation.

For processing the covariates we used a combination of Open Source GIS software, primarily SAGA GIS [28], R packages raster [41], sp [42], GSIF and GDAL [43] for reprojecting, mosaicking and merging tiles. SAGA GIS and GDAL were found to be highly suitable for processing large data as parallelization of computing was relatively easy to implement.

We updated the 1 km global soil mask map using the most detailed 30 m resolution global land cover map from 2010. This was combined with the global water mask [44] and the global sea mask map based on the SRTM DEM [45] to produce one consistent global soil mask that includes all land areas, expect for: (a) fresh water bodies such as lakes and rivers, and (b) permanent ice.

### Spatial prediction framework

**Fig 5:**
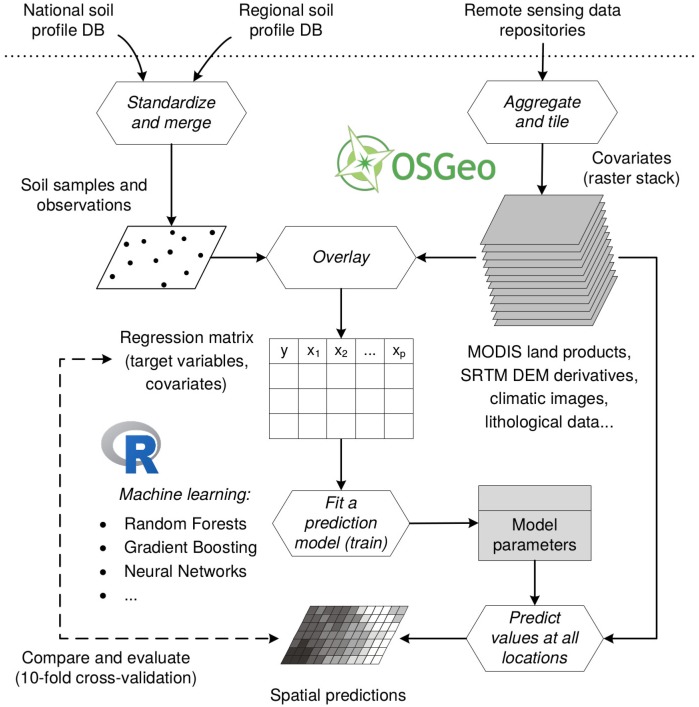
The (data-driven) statistical framework used for generating SoilGrids. SoilGrids are primarily based on publicly released soil profile compilations, NASA’s MODIS and SRTM data products and Open Source software compiled with the ATLAS library: R (including contributed packages), and Open Source Geospatial Foundation (OSGeo) supported software tools.

Spatial prediction, i.e. fitting of models and generation of maps, was fully implemented via the R environment for statistical computing. The process of generating SoilGrids predictions consists of four main steps (see Fig 5):

- overlay points and covariates and prepare regression matrix,
- fit spatial prediction models,
- apply spatial prediction models using tiled raster stacks (covariates),
- assess accuracy using cross-validation.

For practical purposes, we implemented these steps separately for each of the following groups of soil variables:

- WRB soil groups and USDA soil suborders were modelled using ensemble models based on nnet::multinom (which fits multinomial log-linear models via neural networks) [46] and ranger::ranger (fits random forest) functions [47]. We mapped probabilities of occurrence for each individual soil class (118 probability maps for WRB and 67 for USDA),
- Soil properties (organic carbon, bulk density, CEC, pH, soil texture fractions and coarse fragments) were modelled as 3D variables using an ensemble of ranger::ranger and xgboost::xgboost (fits Gradient Boosting Tree) [48]. Soil depth is used as a covariate, so that the resulting models predict values of a target variable for any given depth, i.e. in 3D,
- Depth to bedrock was also modelled using ranger::ranger and xgboost::xgboost functions, but the output is a 2D map.

To optimize the model tuning parameters we consistently used the caret::train function [49], which is also suited for big data. The fine-tuning of the parameters is summarized in the following three steps:

1. Randomly subset the regression matrix to e.g. 15,000 observations (usually 5–10% of the total size),
2. Fit and validate a list of models for a combination of tuning parameters,
3. Select the optimal parameters (i.e. those that produce the lowest RMSE using repeated cross-validation) and fit the final model using all observations.

Models for WRB and USDA classes are defined as:

R> TAXNWRB ~ DEMMRG5 + SLPMRG5 + … + ASSDAC3

where DEMMRG5 + SLPMRG5 + … + ASSDAC3 are the covariate layers, TAXNWRB is the observed taxonomic class in the WRB system (target variable). An example of a soil property model is given by:

R> ORCDRC ~ Depth + DEMMRG5 + … + ASSDAC3

where DEMMRG5 + … + ASSDAC3 are the covariate layers, ORCDRC is the value of organic carbon observed (target variable), and Depth is the sampling / observation depth.

For each variable we fitted a separate model and merged predictions from at least two models to minimize overshooting effects [50]. The merging of predictions is done by using the average model accuracy estimated during the fine-tuning of model parameters, i.e. as a weighted average [50]:

$$\bar{f}\left(x\right)=\frac{\sum_{k=1}^{M}w_{k}\cdot f_{k}\left(x\right)}{\sum_{k=1}^{M}w_{k}},\;\;w_{k}=\frac{1}{\sigma_{k,\text{CV}}^{2}}$$ (2)

where $\bar{f}\left(x\right)$ is the final ensemble prediction, *M* is the number of models, *w*_*k*_ is the model weight and $\sigma_{k,\text{CV}}^{2}$ is the model squared prediction error obtained using cross-validation. In practice, both ranger::ranger and xgboost::xgboost report about the same error in most cases, hence the final prediction is often close to the unweighted average.

We also applied post-processing, mainly to remove artifacts: in the case of soil classes, we filter out all classes theoretically impossible to occur in a given area, such as Gypsisols in arctic climatic zones, using a simple soil-climate matrix (documented on the project github). For texture fractions we also applied a standardization function to ensure that all predictions are between 0 and 100, and that the fractions sum up to 100%, e.g.:

$${\text{Sand}}_{c}\left[\%\right]=\frac{\text{Sand}}{\left(\text{Sand}+\text{Silt}+\text{Clay}\right)}\cdot 100$$ (3)

where Sand_c_ is the corrected sand content.

SoilGrids can be considered as a Big Data project, especially in terms of data volumes and variety. The total size of all input and output data used to generate SoilGrids exceeds 30 TiB, so that a first step in preparing SoilGrids250m was to obtain a Synology 12-Bay NAS storage server with 60 TiB space. Handling such a large data set presented major challenges considering computational complexity and network bandwidth limitations. To optimize computing performance, especially spatial overlay, model fitting, predictions and export of predictions, we used exclusively parallelized versions of functions. For prediction, parallelization is already implemented internally via the ranger or xgboost software; for other processes we primarily used the snowfall package [51].

All processing was implemented on a single dedicated high performance server with 256 GiB RAM, 8 TiB hard disk space, 48 cores (Intel Xeon 2xE5-2690v3 24c/48t 2.6–3.5 GHz) and running on Ubuntu 15.10 (Willy Werewolf) OS and R-cran 3.2.3 using ATLAS (Automatically Tuned Linear Algebra Software) 3.11.38 library. Even after parallelization, producing predictions for all soil variables and all depths took 10+ days of continuous computing, i.e. about 12 thousand CPU hours (about 90% of the computing time is invested in generating predictions). Because the current system is fully scalable, the next update of SoilGrids could be completed in shorter time frames, e.g. by boosting the number of computer cores, although this might also greatly increase the production costs.

### The tiling system

For tiling, we used the Equi7 Grid system [52] which splits the global land mass into seven separate planar grids (Europe and Asia are split into two land masses with some small overlap). The Equi7 Grid system was selected for several practical reasons [52]:

1. The projections of the Equi7 Grid are equidistant and hence suitable for various geographic analyses, especially for derivation of buffer distances and for hydrological DEM modeling, i.e. to derive all DEM-based soil covariates,
2. Areal and shape distortions stemming from the Equi7 Grid projection are relatively small, yielding a small grid oversampling factor,
3. The Equi7 Grid systems ensures an efficient raster data storage while suppressing inaccuracies during spatial transformation. Especially for high-resolution global data, these are important features.

The global soil mask at 250 m resolution contains about 1.6 billion pixels (Africa: 330 million, Europe: 110 million, North America: 230 million, South America: 210 million, Antartica: 0.05 million, Oceania: 140 million, Asia: 360 million). We provide the final outputs in both the Equi7 Grid system and in geographical WGS84 coordinates. Final global mosaics in the WGS84 system were produced by reprojecting all pixels using GDAL warp and translate functions [43]. The ground resolution of 250 m corresponds to a geographical resolution of 1/480 decimal degrees. An image representing the whole world at this resolution comprises 172k columns and 72k rows.

Final predictions are available both as mosaics and as 1° tiles (16,360 tiles to represent the world land mask); tiles are considered more suitable for users interested in regional and national data, and mosaics (at resolutions of 20 km, 1 km and 250 m) are deemed suitable for global modellers.

### Accuracy assessment

For accuracy assessment of both numeric and categorical variables we used 10–fold repeated cross-validation. Each model is re-fitted 10 times using 90% of the data and predictions derived from the fitted models are compared with observations of the remaining 10%. For each of the 14 numeric soil properties we derived the coefficient of determination (*R*^2^—the amount of variation explained by the model), mean error (ME) and root mean squared error (RMSE). The amount of variation explained by the model is derived as:

$$R^{2}=\left[1-\frac{SSE}{SST}\right]\times 100\%$$ (4)

where *SSE* is the sum of squared errors at cross-validation points and *SST* is the total sum of squares. A coefficient of determination close to 1 indicates a perfect model, i.e. 100% of variation has been explained by the model. Numeric variables with skew distributions were log-transformed prior to modeling and hence for these variables we report the amount of variation explained by the model after log-transformation. Also for the cross-validation correlation plots we used either log or linear scale depending on whether log-transformation was applied.

For predictions of soil WRB and USDA classes we calculated the map purity (0–100%) for the dominant soil class at cross-validation points and weighted kappa metrics [53] as implemented in the psych package. For the predicted probabilities of soil class occurrences (0–1 probability values) we also derived the area under the receiver operating characteristic curve (AUC) and the True Positive Rate (TPR) statistic as implemented in the ROCR package [54, 55]. Values of TPR range from 0 to 1. Values of AUC close to 1 show high prediction performance, while values around 0.5 and below are considered poor.

For soil WRB and USDA classes we also generated global maps of the scaled Shannon Entropy Index using the per-class probability maps [56, 57]:

$$H_{s}\left(x\right)=-\sum_{k=1}^{K}p_{k}\left(x\right)\cdot \log_{K}\left(p_{k}\left(x\right)\right)$$ (5)

where *K* is the number of possible classes, log_*K*_ is the logarithm to base *K* and *p*_*k*_ is probability of class *k*. The scaled Shannon Entropy Index (H_*s*_) is in the range from 0–1, where 0 indicates no ambiguity (one of the *p*_*k*_ equals one and all others are zero) and 1 indicates maximum confusion (all *p*_*k*_ equal $\frac{1}{K}$) [58]. Note that the scaled Shannon Entropy Index should not be confused with classification accuracy assessment: H_*s*_ is an internal accuracy measure derived from the model and not based on comparison of predictions with (cross-)validation data, such as the purity and kappa metrics. For Shannon index of 0 at some location accuracy could still be completely wrong because the soil class at that location could actually be a different one.

## Results

### Model fitting

**Fig 6:**
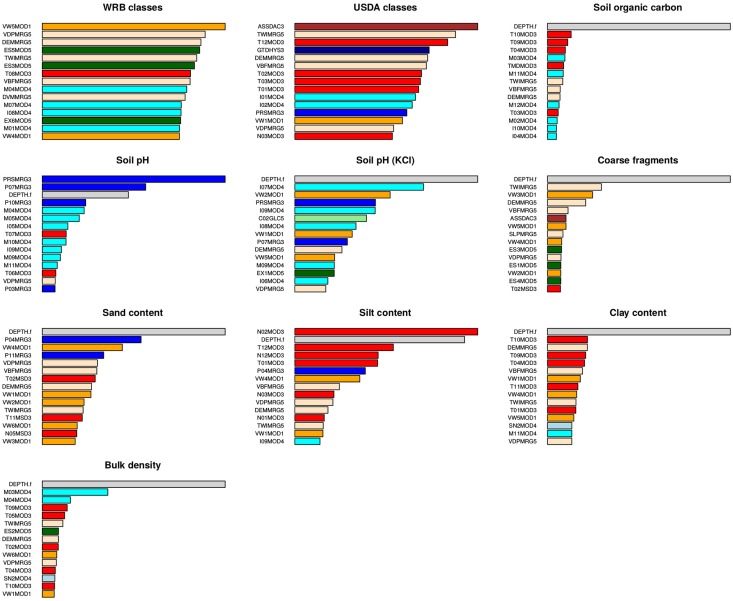
Fitted variable importance plots for target variables. Generated as an average of predictions using the ranger and xgboost packages (for soil types results are based on the ranger model only). DEPTH.f is depth from soil surface, T**MOD3 and N**MOD3 are mean monthly temperatures daytime and nighttime (red color), TWI, DEM, VBF and VDP are DEM-parameters (bisque color), M**MOD4 are mean monthly MODIS NIR band reflectances (cyan color), P**MRG3 are mean monthly precipitation (blue color), E**MOD5 are mean monthly EVI derivatives (dark green color), VW*MOD1 are monthly MODIS Precipitable Water Vapor images (orange color), C**GLC5 are land cover classes (light green color), and ASSDAC3 is the average soil and sedimentary-deposit thickness (brown color).

**Fig 7:**
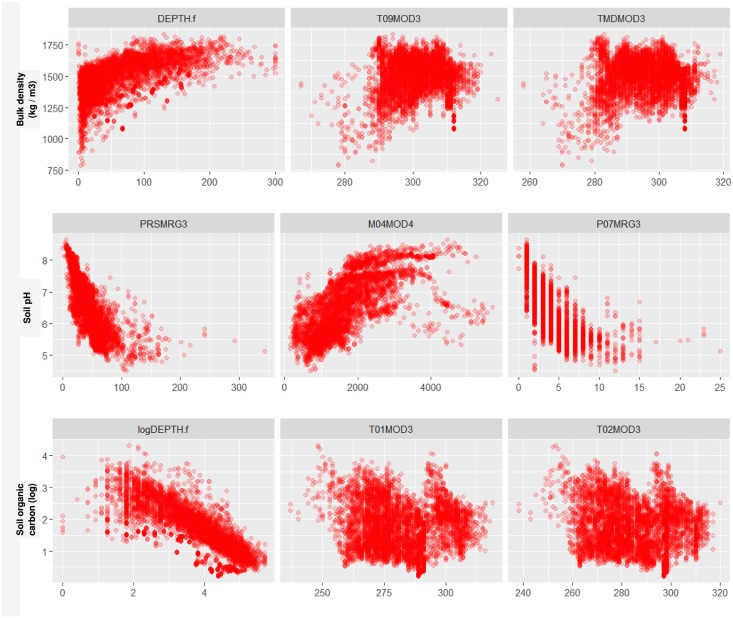
Examples of relationships for target variables and the most important covariates: (top row) bulk density in kg m^−3^, (middle row) soil pH, and (bottom row) soil organic carbon in permilles (on log scale). Plots show target variables and the top three most important covariates as reported by the random forest model. DEPTH.f is the observed depth from soil surface, T09MOD3 is mean monthly temperature for September, TMDMOD3 is mean annual temperature, PRSMRG3 is total annual precipitation, M04MOD4 is mean monthly MODIS NIR band reflectance for April, P07MRG3 is mean monthly precipitation for July, T01MOD3 is mean monthly temperature for January, and T02MOD3 is mean monthly temperature for February.

Summary results of model fitting are given in Figs 6 and 7 and Tables 1 and 2. The ranger package reports model fitting success via the R-square based on Out-of-bag (OOB) samples, i.e. the amount of variation explained by the model, which ranged from a low of 0.59 for coarse fragments to a high of 0.85 for soil pH. R-square estimated using xgboost (derived using repeated cross-validation) was lower, ranging from 0.37 for coarse fragments to 0.60 for soil pH. On average, the two packages report R-square values between 0.4–0.8 with an overall average of 0.60. This number corresponds closely to our results produced using 10–fold cross validation with repeated fitting. Comparing these new results to average R-square values of 0.38 for the original SoilGrids1km predictions reveals a significant improvement of close to + 50%.

**Table 1:** SoilGrids average prediction error for key soil properties based on 10–fold cross-validation. N = “Number of samples used for training”, ME = “Mean Error”, MAE = “Mean Absolute Error”, RMSE = “Root Mean Squared Error” and R-square = “Coefficient of determination” (amount of variation explained by the model). For variables with a skew distribution, such as organic carbon, coarse fragments and CEC, the accuracy statistics are also provided on log-scale^⊗^.

Variable name	N	Min	Max	ME	MAE	RMSE	R-square	RMSE^⊗^	R-square^⊗^
Soil organic carbon (gravimetric)	605,054	0	520	-0.292	10.2	32.8	63.5%	0.715	68.8%
pH index (H_2_O solution)	604,019	2.1	11.0	-0.002	0.4	0.5	83.4%		
Sand content (gravimetric)	616,762	1%	94%	-0.037	9.0	13.1	78.6%		
Silt content (gravimetric)	613,750	2%	74%	0.023	6.7	9.8	79.4%		
Clay content (gravimetric)	625,159	2%	68%	-0.102	6.6	9.5	72.6%		
Coarse fragments (volumetric)	303,139	0%	89%	-0.104	5.5	10.9	55.9%	1.185	64.3%
Bulk density (fine earth fraction)	140,596	250	2870	-1.574	108.3	164.7	75.8%		
Cation-exchange capacity (fine earth fraction)	393,585	0	234	-0.071	5.5	10.3	64.5%	0.483	67.0%
Depth to bedrock (in cm)	1,580,798	0	125,000	-29	678	835	54.0%	1.12	42.8%

**Table 2:** Mapping performance of SoilGrids250m compared to summary results for SoilGrids1km [9]. Amount of variation explained by models (Eq 4), i.e. prediction accuracy for soil types was determined using 10–fold cross-validation. GSIF = “Global Soil Information Facilities”.

Variable name	Type	Units	GSIF code	Amount of var. explained (SoilGrids1km)	Amount of var. explained (SoilGrids250m)	Relative improvement
Soil organic carbon	3D	g kg^−1^	ORCDRC	22.9%	68.8%	200%
pH index (H_2_O solution)	3D	10^−1^	PHIHOX	50.5%	83.4%	65%
Sand content (gravimetric)	3D	kg kg^−1^	SNDPPT	23.5%	78.6%	234%
Silt content (gravimetric)	3D	kg kg^−1^	SLTPPT	34.9%	79.4%	127%
Clay content (gravimetric)	3D	kg kg^−1^	CLYPPT	24.4%	72.6%	198%
Coarse fragments (volumetric)	3D	cm^3^ cm^−3^	CRFVOL	-	64.3%	-
Bulk density (fine earth fraction)	3D	kg m^−3^	BLD	31.8%	75.8%	138%
Cation-exchange capacity (fine earth fraction)	3D	cmol + /kg	CEC	29.4%	67.0%	128%
Depth to bedrock	2D	cm	BDT	-	42.8%	-

The train function of the package caret usually picked a relatively high Mtry parameter (number of variables randomly sampled as candidates at each split) as optimal for soil properties: the optimized values ranged from 18 for coarse fragments to 22 for all other soil properties. Higher Mtry is recommend for cases where the number of covariates is large and multiple variables influence the target variables with equal importance [59]. For the Gradient Boosting Tree method, train always selected the same combination of tuning parameters for all soil properties: nrounds = 100, max_depth = 3, eta = 0.4, gamma = 0, colsample_bytree = 0.8 and min_child_weight = 1. This may be because we limited the combinations of tuning parameters to 10 to speed up processing speed. Higher values for xgboost tuning parameters are indicative of higher-level complexity of the model: many relationships between soil properties and covariates are non-linear and a greater number of splits is possibly required to represent this complexity.

Fig 6 shows the top 15 soil covariates for each target variable. This indicates that, for example, spatial pattern of soil pH is primarily influenced by precipitation and surface reflectance (MODIS Medium-Infrared band 6 for months April and May especially). Also, for most variables depth emerges as the most important covariate, especially for soil organic carbon, bulk density and coarse fragments. For soil types and soil textures, DEM-parameters, i.e. soil forming factors of relief, especially flow-based DEM-indices, emerge as second-most dominant covariates. These results largely correspond with conventional soil survey knowledge (surveyors have been using relief as a key guideline to delineate soil bodies for decades), but it is encouraging to have these findings supported by statistical modeling of real data on a global scale.

Although lithology is not in the list of top 15 most important predictors, spatial patterns of lithologic classes can often be distinctly recognized in the output predictions. This is especially true for soil texture fractions and coarse fragments. In general, for predicting soil chemical properties, climatic variables (especially precipitation) and surface reflectance seem to be the most important, while for soil classes and soil physical properties it is a combination of relief, vegetation dynamics and parent material.

Fig 7 shows some individual relationships between target variables and several of the most important covariates. For soil pH we observe that the relationship with total annual rainfall is close to linear; for soil organic carbon and depth the relationship is linear on a log-log scale. Many such individual correlations can also be interpreted and understood in terms of pedologic knowledge. For example, higher MIR reflectance may be associated with high concentration of salts in soil and hence higher pH; higher rainfall and cooler climates often result in higher organic carbon content because the speed of organic matter accumulation is higher than the speed of decomposition. For the majority of soil variables, however, relationships are not clearly linear and often many soil covariates are equally important.

We have also investigated possibilities for using kriging of residuals to improve predictions of soil properties. Because the majority of spatial variation has been explained by covariates and machine learning models, it appears that no significant spatial autocorrelation structure can be observed for residuals (i.e. almost all variograms show pure nugget effect structure) at distances < 300 km for almost all continents and all variables. Although locally, where the point density is high, kriging of residuals could still be beneficial for mapping of CEC and depth to bedrock, overall kriging of residuals for global land mass does not seem to be necessary nor is it practical to implement for billions of pixels: it would only marginally improve the accuracy of predictions at high computing costs.

### Accuracy assessment

**Fig 8:**
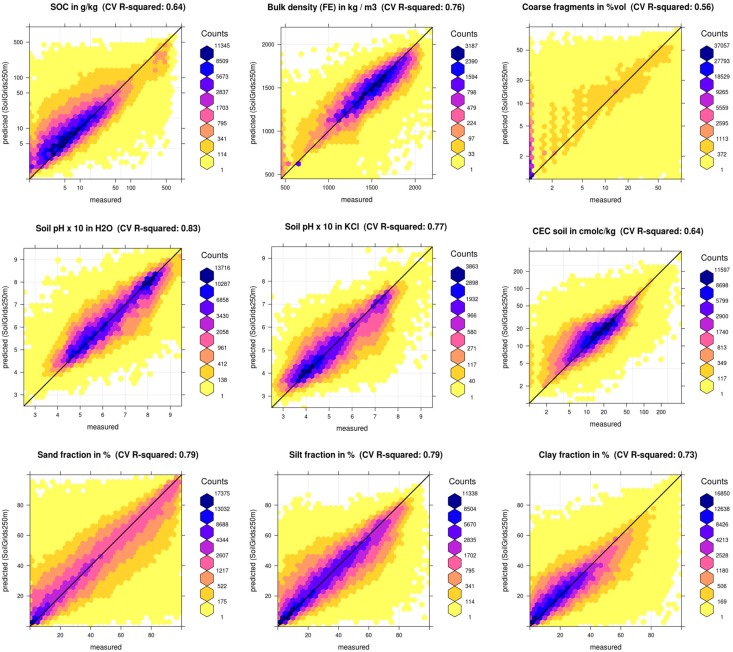
Correlation (density) plots produced as a result of 10–fold cross-validation. See also Table 1 for more details.

Table 1 shows summary results of cross-validation for soil properties (global assessment). In all cases there is no large overestimation of values, although for organic carbon and CEC the models seem to somewhat under-estimate the overall mean. For log-transformed variables we applied the accuracy assessment in the log-transformed space which yields asymmetric prediction intervals after back-tranformation. For example, predictions for organic carbon are ±0.715 in log-space, which means that the 90% probability prediction interval for a case where the soil organic carbon prediction equals 20‰ (2%) is 6–65‰; for a case where the soil organic carbon prediction equals 150‰ it is 46–485‰. Prediction intervals are hence still fairly wide, which might make SoilGrids of limited usability for detailed spatial modeling e.g. at farm level. Note also that because there is significant spatial clustering of the training points, it is possible that the validation results might be somewhat more optimistic than if we had validated predictions by using points collected following some (objective) probability sampling, as described in Brus et al. [60]. On the other hand, the cross-validation results do not show any serious systematic over- or underestimation (ME close to zero), which is also visible from the correlation plots (Fig 8).

Table 2 shows results for SoilGrids250m in comparison with the previous system at 1 km resolution. Improvements in average RMSE are between 30–80% and can largely be attributed to the use of machine learning algorithms in place of multiple linear regression, but also to investments in preparing finer resolution covariates and additional and improved soil profile data.

The most challenging variables to model with this set of covariates are coarse fragments and depth to bedrock, although in no case is the R-square < 50%. Nevertheless, the RMSE is still relatively high in comparison to many local soil mapping projects. Users should thus be aware that the uncertainty levels are still relatively high. There are also still problems with overestimation of low values, clearly visible for example in the case of soil organic carbon content. Overall, predictions for most properties are unbiased, i.e. most predictions are fairly symmetric around the 1:1 line (Fig 8).

For soil classes, out-of-bag average prediction accuracy, reported by the packages, was between 20–28% for the WRB classification system and between 34–48% for the USDA system. The 10–fold cross-validation results showed that the weighted kappa for WRB classes is 42%, with map purity 28%; for USDA classes the kappa is 57%, while the map purity is 48%. Although WRB classes seem to be somewhat more challenging to model than USDA suborders, this comparison should be considered within the context of: (a) the number of classes, and (b) similarity between classes. The WRB classification contains about two times more classes than USDA suborders, and many WRB classes with highest confusion fall in taxonomically similar groups. Further evaluation of classification accuracy has shown that, at the level of WRB soil groups, map purity jumps to 60%, i.e. it becomes comparable to the USDA system. Remaining WRB soil groups with map purity < 50% are Planosols, Phaeozems and Ferrasols.

**Fig 9:**
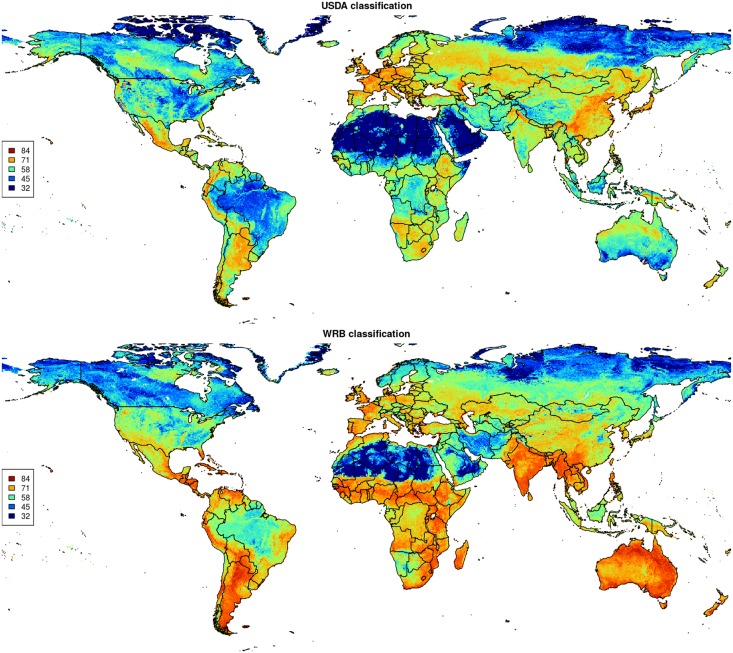
Maps of scaled Shannon Entropy index (Eq 5) for USDA and WRB soil classification maps.

**Fig 10:**
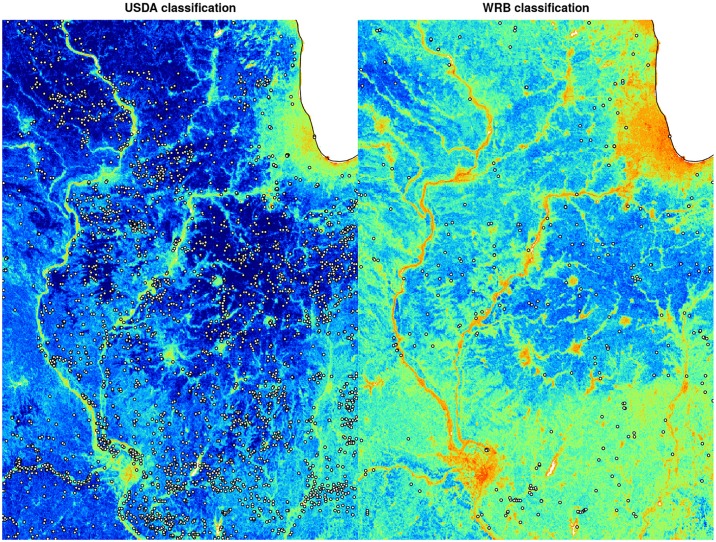
Example of scaled Shannon Entropy index for USDA and WRB soil classification maps with a zoom in on USA state Illinois near the city of Chicago. This figure uses the same legend as used in Fig 9.

A more detailed assessment of prediction accuracy derived using the ROCR package, i.e. per each individual class, shows that the average TPR is about 0.93 for USDA soil suborders (Table 3), and about 0.90 for WRB classes (Table 4). Also maps of the scaled Shannon Entropy index (Fig 9) indicate that produced soil class maps for USDA soil classification system are less uncertain than for the WRB system: WRB classification is critically uncertain for Australia and India, parts of Africa and highlands of Latin America. Maps of uncertainty closely reflect extrapolation areas and could be potentially very useful for planning new soil surveys aimed at mapping soil types. For example, Fig 10 shows that the highest confusion (lowest prediction accuracy) is systematically connected with distribution of river valleys, urban areas and hill-slopes.

**Table 3:** Classification accuracy for predicted USDA class probabilities based on 10–fold cross-validation, ordered according to number of occurrences. ME = “Mean Error”, TPR = “True Positive Rate”, AUC = “Area Under Curve”, N = “Number of occurrences”, USDA = “United States Department of Agriculture” soil classification system. The 1st and 2nd most probable classes are taken from the confusion matrix.

Name	ME (%)	TPR	AUC	N	1st class	2nd class
Udalfs	0.0	0.88	0.93	6326	Udalfs	Udults
Udults	0.0	0.91	0.95	4997	Udults	Udalfs
Udolls	0.1	0.91	0.93	3901	Udolls	Udalfs
Ochrepts	0.1	0.89	0.91	2720	Ochrepts	Udalfs
Aqualfs	0.0	0.89	0.91	2594	Aqualfs	Udalfs
Aquolls	0.1	0.89	0.90	2450	Udolls	Aquolls
Udox	0.0	0.93	0.95	2229	Ustox	Udox
Ustolls	-0.2	0.95	0.97	2042	Ustolls	Borolls
Borolls	0.1	0.97	0.98	2029	Borolls	Albolls
Ustox	0.1	0.93	0.95	2024	Ustox	Udox
Orthents	0.1	0.88	0.89	1911	Orthents	Udults
Aquepts	0.0	0.87	0.88	1734	Aquolls	Aquepts
Psamments	0.1	0.90	0.92	1725	Psamments	Udults
Fluvents	0.1	0.84	0.85	1579	Udults	Udalfs
Orthods	0.1	0.97	0.98	1538	Orthods	Ochrepts
Udepts	0.1	0.90	0.91	1429	Udepts	Udults
Aquents	-0.1	0.84	0.85	1342	Aquepts	Udalfs
Ustalfs	-0.1	0.95	0.96	1332	Ustalfs	Ustolls
Xerolls	0.0	0.97	0.98	1319	Xerolls	Xeralfs
Argids	-0.1	0.98	0.99	907	Argids	Xerolls
Turbels	0.1	0.99	1.00	787	Turbels	Orthels
Orthels	0.0	0.97	0.98	648	Ochrepts	Orthels
Xeralfs	-0.3	0.97	0.98	615	Xeralfs	Xerolls
Usterts	-0.2	0.97	0.98	590	Usterts	Ustolls
Albolls	-0.2	0.92	0.93	589	Borolls	Aquolls
Xerepts	-0.3	0.99	0.99	588	Xerepts	Xeralfs
Arents	-0.2	0.99	0.99	554	Arents	Ustox
Aquults	-0.2	0.94	0.94	380	Udults	Aquults
Cambids	-0.1	0.99	0.99	362	Cambids	Argids
Humults	-0.1	0.98	0.98	348	Humults	Udults
Hemists	-0.2	0.93	0.93	347	Ochrepts	Hemists
Torrox	-0.3	0.98	0.99	334	Ustox	Torrox
Saprists	-0.4	0.93	0.93	319	Saprists	Udalfs
Histels	-0.3	0.99	0.99	302	Histels	Turbels
Aquods	0.0	0.94	0.94	301	Orthods	Udults
Calcids	-0.1	0.98	0.99	301	Argids	Calcids
Ustults	0.0	0.99	0.99	286	Ustults	Ustalfs
Fibrists	0.0	0.96	0.97	250	Fibrists	Udults
Udands	0.0	0.99	0.99	234	Udands	Udox
Xerands	0.0	0.99	0.99	231	Xerands	Xerolls
Aquerts	-0.2	0.95	0.95	226	Aqualfs	Udalfs
Xererts	0.0	0.96	0.96	184	Xererts	Xerolls
Uderts	0.0	0.95	0.96	177	Udults	Uderts
Ustepts	0.0	0.97	0.97	175	Ustolls	Ustepts
Cryands	0.0	0.99	0.99	161	Cryands	Ochrepts
Cryepts	0.0	0.98	0.98	150	Ochrepts	Cryepts
Humods	0.0	0.92	0.92	149	Orthents	Orthods
Cryods	-0.1	0.99	1.00	133	Orthods	Cryods
Torrerts	-0.1	0.98	0.98	106	Ustolls	Torrerts
Cryolls	-0.2	0.99	0.99	79	Borolls	Cryolls
Gelods	-0.7	1.00	1.00	78	Turbels	Gelods
Gypsids	-0.1	0.99	0.99	70	Argids	Gypsids
Vitrands	-0.3	0.98	0.98	62	Vitrands	Ochrepts
Torrands	-0.3	0.99	0.99	60	Xerolls	Torrands
Durids	-0.3	0.99	0.99	59	Argids	Xerolls
Xerults	-0.3	0.97	0.97	53	Xeralfs	Humults
Rendolls	-0.5	0.93	0.93	41	Udalfs	Ochrepts
Salids	-0.6	0.94	0.94	37	Argids	Fluvents
Cryalfs	-0.7	1.00	1.00	32	Ochrepts	Borolls
Folists	-0.5	0.99	0.99	30	Orthods	Cryods
Gelands	-1.0	0.97	0.97	26	Gelods	Turbels
Perox	-0.7	0.99	0.99	21	Udults	Perox
Aquands	-0.7	0.98	0.98	19	Xerolls	Udands
Ustands	-0.9	1.00	1.00	17	Ustalfs	Orthents
Aquox	-1.0	0.98	0.98	16	Udults	Udox
Cryids		0.99	0.99	8	Argids	Borolls
Gelepts		0.83	0.83	6	Ochrepts	Turbels

**Table 4:** Classification accuracy for predicted WRB class probabilities based on 10–fold cross-validation, ordered according to number of occurrences. ME = “Mean Error”, TPR = “True Positive Rate”, AUC = “Area Under Curve”, N = “Number of occurrences”, WRB = “World Reference Base” soil classification system. The 1st and 2nd most probable classes are taken from the confusion matrix.

Name	ME (%)	TPR	AUC	N	1st class	2nd class
Haplic Cambisols	0.1	0.78	0.81	5619	Haplic Cambisols	Haplic Cambisols (Dystric)
Haplic Luvisols	0.1	0.86	0.88	2975	Haplic Luvisols	Haplic Cambisols
Haplic Acrisols	0.0	0.93	0.94	2607	Haplic Acrisols	Haplic Ferralsols
Haplic Ferralsols	-0.1	0.94	0.95	1887	Haplic Ferralsols	Haplic Acrisols
Haplic Fluvisols	-0.1	0.88	0.89	1776	Haplic Fluvisols (Calcaric)	Haplic Fluvisols (Eutric)
Haplic Calcisols	0.0	0.93	0.94	1745	Haplic Calcisols	Calcaric Regosols
Haplic Kastanozems	0.0	0.96	0.97	1718	Haplic Kastanozems	Haplic Luvisols
Gleyic Luvisols	0.0	0.92	0.93	1686	Albic Luvisols	Gleyic Luvisols
Aric Regosols	-0.2	0.91	0.92	1488	Calcaric Regosols	Haplic Leptosols
Haplic Chernozems	0.1	0.96	0.97	1394	Haplic Chernozems	Haplic Kastanozems
Albic Luvisols	-0.1	0.94	0.95	1389	Gleyic Luvisols	Haplic Luvisols
Calcaric Regosols	-0.2	0.92	0.93	1379	Aric Regosols	Haplic Leptosols
Haplic Podzols	0.1	0.96	0.97	1359	Haplic Podzols	Haplic Cambisols
Haplic Cambisols (Dystric)	0.0	0.90	0.91	1334	Haplic Cambisols	Haplic Podzols
Haplic Cambisols (Calcaric)	-0.1	0.92	0.92	1173	Haplic Cambisols	Haplic Calcisols
Haplic Phaeozems	-0.1	0.90	0.91	1114	Haplic Phaeozems	Haplic Chernozems
Haplic Lixisols	0.0	0.92	0.93	1094	Haplic Lixisols (Chromic)	Haplic Lixisols
Haplic Leptosols	0.0	0.91	0.92	1092	Haplic Leptosols	Haplic Leptosols (Eutric)
Haplic Gleysols	0.0	0.88	0.89	1054	Haplic Gleysols (Eutric)	Haplic Cambisols
Haplic Vertisols	0.1	0.93	0.93	1040	Haplic Vertisols (Eutric)	Haplic Vertisols
Haplic Arenosols	0.1	0.91	0.92	935	Haplic Arenosols	Haplic Cambisols
Ferralic Arenosols	0.1	0.96	0.97	920	Ferralic Arenosols	Haplic Ferralsols
Haplic Cryosols	0.0	0.99	1.00	884	Haplic Cryosols	Haplic Cambisols
Haplic Cambisols (Eutric)	0.1	0.84	0.85	857	Haplic Cambisols	Haplic Luvisols
Haplic Alisols	0.1	0.94	0.95	827	Haplic Acrisols	Haplic Cambisols
Luvic Phaeozems	-0.4	0.90	0.91	741	Luvic Phaeozems	Haplic Luvisols
Rendzic Leptosols	0.0	0.94	0.95	695	Haplic Cambisols	Rendzic Leptosols
Haplic Fluvisols (Calcaric)	0.0	0.94	0.94	692	Haplic Fluvisols	Haplic Calcisols
Petric Calcisols	0.1	0.97	0.98	679	Petric Calcisols	Haplic Calcisols
Haplic Regosols (Eutric)	0.1	0.86	0.87	677	Haplic Cambisols	Haplic Luvisols
Lithic Leptosols	0.0	0.93	0.93	655	Haplic Ferralsols	Haplic Acrisols
Umbric Gleysols	0.0	0.91	0.92	621	Mollic Gleysols	Calcic Gleysols
Mollic Gleysols	0.0	0.91	0.91	575	Umbric Gleysols	Calcic Gleysols
Haplic Vertisols (Eutric)	0.1	0.95	0.95	568	Haplic Vertisols	Haplic Kastanozems
Haplic Gypsisols	0.1	0.98	0.98	565	Haplic Gypsisols	Aric Regosols
Haplic Solonetz	0.1	0.92	0.92	539	Gleyic Solonetz	Solodic Planosols
Calcic Gleysols	0.0	0.90	0.91	514	Umbric Gleysols	Mollic Gleysols
Haplic Nitisols (Rhodic)	0.1	0.94	0.95	492	Haplic Ferralsols	Haplic Acrisols
Haplic Fluvisols (Eutric)	0.1	0.91	0.91	465	Haplic Fluvisols	Haplic Ferralsols
Haplic Lixisols (Chromic)	0.0	0.97	0.97	441	Haplic Lixisols	Haplic Ferralsols
Calcic Vertisols	-0.1	0.93	0.93	437	Calcic Vertisols	Haplic Vertisols
Calcic Kastanozems	-0.1	0.95	0.95	415	Haplic Kastanozems	Haplic Luvisols
Leptic Regosols	0.1	0.96	0.96	404	Petric Calcisols	Haplic Luvisols
Haplic Luvisols (Chromic)	-0.1	0.93	0.93	396	Haplic Luvisols	Haplic Ferralsols
Haplic Solonchaks	0.1	0.95	0.95	383	Haplic Solonchaks (Sodic)	Haplic Solonchaks
Luvic Chernozems	-0.1	0.93	0.93	377	Haplic Kastanozems	Luvic Phaeozems
Acric Ferralsols	0.0	0.97	0.98	371	Haplic Ferralsols	Acric Ferralsols
Fibric Histosols	0.0	0.96	0.96	371	Fibric Histosols	Haplic Acrisols
Calcic Luvisols	0.0	0.92	0.92	369	Haplic Cambisols	Haplic Luvisols
Calcic Chernozems	0.0	0.94	0.94	358	Calcic Chernozems	Haplic Cambisols
Aluandic Andosols	0.0	0.97	0.97	341	Aluandic Andosols	Haplic Cambisols
Luvic Calcisols	0.0	0.95	0.95	322	Haplic Calcisols	Haplic Kastanozems
Protic Arenosols	0.0	0.99	0.99	322	Protic Arenosols	Haplic Leptosols
Haplic Albeluvisols	-0.1	0.97	0.97	321	Haplic Albeluvisols	Haplic Cambisols
Mollic Solonetz	-0.1	0.94	0.94	312	Gleyic Solonetz	Haplic Kastanozems
Haplic Acrisols (Ferric)	-0.1	0.95	0.95	310	Haplic Acrisols	Haplic Cambisols
Haplic Planosols (Eutric)	0.0	0.89	0.89	310	Haplic Podzols	Haplic Acrisols
Haplic Gleysols (Eutric)	-0.1	0.93	0.93	306	Haplic Gleysols	Haplic Acrisols
Ferralic Cambisols	0.0	0.93	0.93	301	Haplic Ferralsols	Haplic Acrisols
Cryic Histosols	-0.1	0.99	0.99	299	Cryic Histosols	Haplic Cryosols
Gleyic Solonetz	-0.2	0.94	0.94	282	Mollic Solonetz	Haplic Solonetz
Haplic Cambisols (Humic)	0.0	0.92	0.93	273	Haplic Cambisols	Haplic Acrisols
Leptic Phaeozems	0.1	0.97	0.97	267	Leptic Phaeozems	Haplic Luvisols
Haplic Regosols (Dystric)	0.0	0.87	0.87	262	Haplic Cambisols	Haplic Acrisols
Haplic Leptosols (Eutric)	0.0	0.93	0.93	261	Haplic Leptosols	Haplic Calcisols
Acric Plinthosols	0.1	0.97	0.97	251	Haplic Acrisols	Haplic Ferralsols
Hemic Histosols	0.0	0.97	0.97	250	Hemic Histosols	Albic Luvisols
Endogleyic Cambisols	0.0	0.87	0.87	249	Haplic Cambisols	Haplic Acrisols
Haplic Cambisols (Chromic)	0.0	0.93	0.93	242	Haplic Cambisols	Haplic Ferralsols
Vertic Cambisols	0.1	0.88	0.88	241	Haplic Ferralsols	Haplic Cambisols
Leptic Luvisols	0.2	0.96	0.97	229	Haplic Luvisols	Leptic Phaeozems
Solodic Planosols	0.0	0.96	0.96	222	Haplic Solonetz	Haplic Kastanozems
Hypoluvic Arenosols	0.0	0.96	0.96	205	Hypoluvic Arenosols	Haplic Arenosols
Leptic Cambisols	0.1	0.94	0.94	199	Haplic Luvisols	Petric Calcisols
Umbric Ferralsols	0.1	0.96	0.96	192	Haplic Ferralsols	Haplic Acrisols
Gleyic Podzols	0.1	0.95	0.95	176	Gleyic Podzols	Haplic Acrisols
Turbic Cryosols	0.0	0.99	1.00	168	Haplic Cryosols	Turbic Cryosols
Vitric Andosols	0.1	0.97	0.97	166	Haplic Cambisols	Aluandic Andosols
Haplic Acrisols (Humic)	0.0	0.96	0.96	164	Haplic Acrisols	Haplic Cambisols
Haplic Fluvisols (Arenic)	0.0	0.98	0.98	163	Haplic Fluvisols	Ferralic Arenosols
Stagnic Luvisols	0.0	0.93	0.93	163	Haplic Cambisols	Haplic Luvisols
Mollic Leptosols	0.0	0.90	0.90	162	Petric Calcisols	Haplic Leptosols
Haplic Acrisols (Alumic)	0.0	0.98	0.98	156	Haplic Acrisols	Haplic Ferralsols
Plinthic Acrisols	0.0	0.94	0.94	152	Haplic Acrisols	Plinthic Acrisols
Calcic Solonetz	0.0	0.93	0.93	149	Haplic Calcisols	Haplic Kastanozems
Haplic Ferralsols (Xanthic)	0.0	0.96	0.96	146	Haplic Ferralsols	Haplic Acrisols
Vertic Luvisols	-0.1	0.93	0.94	140	Haplic Cambisols	Haplic Luvisols
Haplic Lixisols (Ferric)	-0.1	0.96	0.96	134	Haplic Lixisols	Haplic Acrisols
Mollic Vertisols	0.0	0.96	0.96	133	Mollic Vertisols	Haplic Cambisols
Haplic Solonchaks (Sodic)	-0.1	0.97	0.97	130	Haplic Solonchaks	Haplic Arenosols
Sapric Histosols	-0.1	0.90	0.90	128	Haplic Cambisols	Fibric Histosols
Haplic Ferralsols (Rhodic)	-0.1	0.96	0.96	125	Haplic Ferralsols	Haplic Acrisols
Calcic Gypsisols	-0.4	0.96	0.96	124	Calcaric Regosols	Haplic Calcisols
Haplic Cambisols (Sodic)	-0.1	0.98	0.98	120	Haplic Cambisols	Ferralic Arenosols
Haplic Calcisols (Sodic)	-0.4	0.98	0.98	115	Haplic Calcisols	Haplic Cambisols
Haplic Fluvisols (Dystric)	-0.2	0.93	0.93	107	Haplic Fluvisols	Haplic Ferralsols
Haplic Gleysols (Dystric)	-0.3	0.91	0.91	100	Haplic Gleysols	Haplic Ferralsols
Gypsic Solonchaks	-0.4	0.98	0.98	98	Haplic Gypsisols	Calcaric Regosols
Haplic Luvisols (Ferric)	-0.2	0.95	0.95	98	Haplic Luvisols	Haplic Lixisols
Haplic Arenosols (Calcaric)	-0.3	0.94	0.94	97	Haplic Arenosols	Haplic Calcisols
Umbric Albeluvisols	-0.2	0.99	1.00	97	Umbric Albeluvisols	Haplic Albeluvisols
Alic Nitisols	-0.1	0.98	0.98	70	Haplic Acrisols	Alic Nitisols
Haplic Andosols	-0.2	0.93	0.93	67	Aluandic Andosols	Haplic Luvisols
Haplic Planosols (Dystric)	0.0	0.92	0.92	62	Ferralic Arenosols	Haplic Ferralsols
Luvic Stagnosols	0.0	0.99	0.99	61	Haplic Cambisols	Gleyic Luvisols
Haplic Umbrisols	0.0	0.93	0.93	57	Haplic Cambisols	Haplic Acrisols
Albic Arenosols	-0.1	0.93	0.93	54	Haplic Acrisols	Haplic Arenosols
Lixic Plinthosols	-0.2	0.94	0.94	49	Haplic Ferralsols	Haplic Acrisols
Leptic Umbrisols	-0.4	0.97	0.97	40	Haplic Luvisols	Haplic Leptosols
Petric Durisols	-0.3	1.00	1.00	39	Petric Durisols	Haplic Phaeozems
Cutanic Alisols	-0.4	0.98	0.98	34	Haplic Cambisols	Cutanic Alisols
Endogleyic Planosols	-0.2	0.93	0.93	34	Haplic Acrisols	Haplic Luvisols
Haplic Regosols (Sodic)	-0.5	0.97	0.97	34	Haplic Vertisols	Leptic Regosols
Luvic Planosols	-0.4	0.88	0.88	29	Haplic Luvisols	Haplic Cambisols
Calcic Histosols	-0.9	0.94	0.94	18	Haplic Acrisols	Haplic Gleysols
Vetic Acrisols	-1.6	0.90	0.90	15	Haplic Acrisols	Haplic Ferralsols
Histic Albeluvisols	-2.3	1.00	1.00	13	Umbric Albeluvisols	Fibric Histosols
Vitric Cryosols	-2.3	1.00	1.00	13	Vitric Cryosols	Haplic Cambisols

In summary, the cross-validation results for predicting class probabilities indicate relatively high correspondence between prediction probabilities and observed soil types, which is also confirmed visually by overlaying observed classes and prediction probabilities. Nevertheless, it appears from Tables 3 and 4 that for some classes, such as Cambisols, Luvisols, Fluvisols and Planosols in the WRB system, and Aquepts, Fluvents and Aquents in the USDA Soil Taxomony system, the confusion of predictions with other classes is still relatively high.

## Discussion

**Fig 11:**
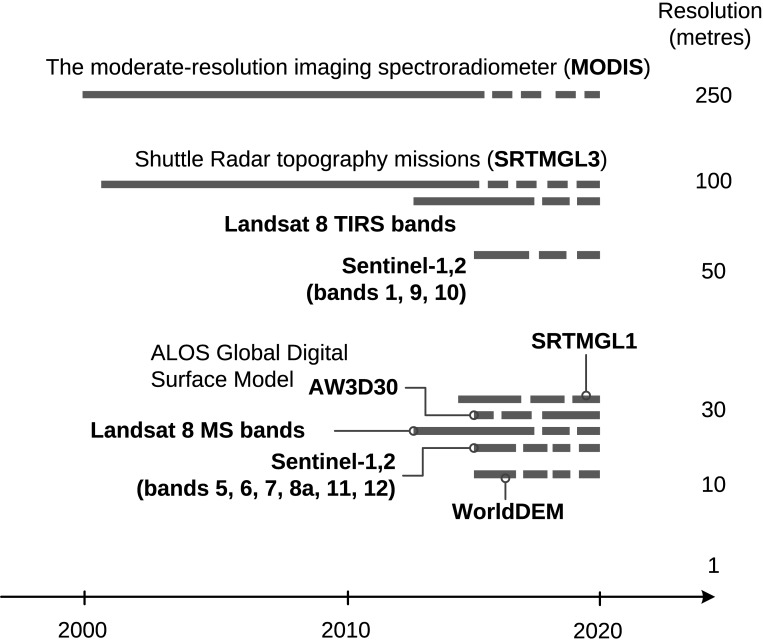
List of some remote sensing data of relevance for global soil mapping projects (i.e. with a near to global coverage and with remote sensing technology of interest to soil mapping). Landsat 8 is part of the Landsat Data Continuity Mission (LDCM) maintained by NASA and the United States Geological Survey (USGS). ALOS Global Digital Surface Model is a product of the Japanese Aerospace Exploration Agency. Sentinel–1,2 is the Earth observation mission developed by the European Space Agency as part of the Copernicus Programme. WorldDEM^™^ is a commercial product distributed by Airbus Defence and Space.

In the following sections we address some remaining discussion points and suggest ways to improve SoilGrids and embark on new research directions. Although we have reached current effective limits imposed by software capabilities and availability of remote sensing data sources, the accuracy of SoilGrids could still be improved. Globally, by adding more covariates based on the most recent remote sensing data (see Fig 11), and locally, by combining global predictions with local prediction models. Global models could be further improved especially by revising (even re-designing) each of the three main components of the system:

- Soil training data,
- Statistical / Machine Learning models, and
- Covariate layers.

### Increasing and improving the quality and quantity of the training data

The most fruitful avenue for improving the current predictions is likely in improving the quality and quantity of soil profile data. ISRIC has invested decades in obtaining, digitizing, cleaning up and standardizing soil profile data. A large portion of these data (about 80,000 unique points) is publicly available via ISRIC’s Web Feature Service WoSIS (http://wfs.isric.org/geoserver/wosis/wfs) [61]; remaining soil profile data sets not publicly available via ISRIC’s WoSIS WFS can be obtained by contacting the corresponding original data providers as listed in the Acknowledgment section. This collection of soil profile data is of similar scope and utility when compared to other international data initiatives in meteorology (e.g. Global Historical Climatology Network) and biodiversity (http://gbif.org).

Although the training data shown in Fig 3 appear to be quite dense, there are still large gaps in terms of representation of the feature space. Tropics, wetlands, semi-arid to hyper-arid areas and mountains are still largely under-represented. There are undoubtedly millions of soil field observations in the world unused for global soil mapping activities that could be collated and used to improve predictions. FAO’s Global Soil Partnership (http://www.fao.org/globalsoilpartnership/) has set as one of its main objectives the preparation of an international compilation of reference soil profiles to help catalyze using soil data for decision making. Hence, there are already some initiatives in this direction.

Harmonization of soil laboratory data and soil descriptive variables is another area that will need to be improved. For example, we had to standardize soil depths for several databases by re-aligning 0 depth to soil surface. Some soil databases only contain information about the mineral soil and put the zero level at the start of the mineral soil. But such soils might have an organic layer as well. Since the thickness of the organic horizon of these soil profiles is not reported, their vertical coordinates could not be corrected. There are many situations like this that require careful analysis of harmonization steps, so that any serious over or under-estimation can be avoided.

It is also fundamentally important that we do not limit ourselves to legacy soil profile data only. The soil science community needs to actively begin investing in collecting new soil profile field observations, especially in the previously mentioned ecological and climatic zones that have been under-sampled. For example, the AfSIS project (http://africasoils.net) has spent already half a decade on collecting new samples for Africa. We believe that there is great potential in undertaking various types of feature space distribution analysis (see e.g. Minasny et al. [62] and Fitzpatrick et al. [63]) and optimizing new sampling of additional soil profiles using, for example, Latin Hypercube sampling principles. By adding only a few hundred new points that are carefully allocated in extrapolation areas, the accuracy of predictions is likely to improve more rapidly than if we double the number of points in areas already well represented. Collection of the new samples could even be implemented via crowd labour or crowdsourcing systems so that also local soil surveyors / enthusiasts could get involved (we are currently testing using MySoil observations contributed by non-specialists, kindly donated to SoilGrids by the British Geological Survey).

### Improving the modeling framework

A major improvement from SoilGrids1km to SoilGrids250m is that we now consistently use machine learning techniques to generate predictions. In the previous version of SoilGrids we used various types of (Generalized) Linear Models in combination with natural splines to model soil property-depth relationships, but this resulted in soil property-depth relationships that were the same across the globe, which is unrealistic and suboptimal. To tackle such problems we now use dominantly tree-based models—random forest and gradient tree boosting—to account for local relationships between soil variables and covariates. Fig 2 (left) shows that, indeed, predictions produced using tree-based models adjust locally to observed values. The current version of SoilGrids is thus, we contend, able to better represent both global and local patterns.

It has already been demonstrated that random forest can outperform linear models, especially in being able to better represent complex non-linear relationships in large data sets [64–66]. Likewise, gradient tree boosting has already won several Kaggle.com competitions (Kaggle is a platform for predictive modeling and analytics competitions on which companies and researchers post their data and statisticians and data miners from all over the world compete to produce the best models). However, tackling the complexities of data size has been a major challenge. In the case of SoilGrids, the regression matrices had up to one million point pairs with over 150 covariates, hence their size and complexity well exceeds what can be handled with desktop computers. Ultimately, we decided to primarily rely on three R packages—caret [49], ranger [47] and xgboost [48]—that have proven to be capable of processing huge raster stacks. By using these three open source packages and a single dedicated server (current costs of about $800 / month) we were able to optimize and fit all models needed to generate SoilGrids within a few hours, and to generate all predictions for the entire world within 12 days.

Machine learning (ML) greatly simplifies model fitting: basically, a soil surveyor does not need to suggest or impose any relationships—the analyst only needs to list a target variable and covariates, and machine learning does the ‘magic’ of optimizing model parameters. On the one hand this is an attractive property because using the ML framework for global soil mapping allows mapping hundreds of soil variables in parallel with little human interaction. On the other hand it has also risks and limitations:

- ML is sensitive to noise and errors in the data. Even a few typos in the input values can result in significant blunders in output maps,
- The computational intensity of ML, when compared to fitting linear models or similar, is an order of magnitude greater. As the number of training points grows, the computational load grows exponentially. At some stage, it becomes currently infeasible and overly expensive to compute predictions using machine learning,
- Extrapolation of models fitted using ML remains risky. Without using pseudo-points to fill-in data gaps in feature space for some parameters, machine learning can potentially produce worse maps (on average for most of the soil mask) than linear models,
- Because the sampling locations are clearly biased towards agricultural areas, and because most of the training points come from the developed world (especially USA), it is very well possible that SoilGrids predictions are significantly biased in undersampled parts of the world. In principle, the best solution to this problem is to continuously add more points from undersampled areas, especially in Africa (tropical soils and wetlands) and the Russian Federation,
- With ML approaches it is difficult to derive spatially explicit measures of the prediction accuracy. We calculated accuracy measures using 10–fold cross-validation, but these are only global measures.
- ML approaches have a high degree of “black box” modeling and it is difficult to incorporate knowledge of soil forming processes in the prediction algorithm. But perhaps we can also learn from ML models by closer inspection and interpretation of how dominant covariates influence soil property and soil class predictions.

Could machine learning put soil mappers out of work? Probably not. Solid knowledge of soil science, spatial statistics and/or geostatistics in projects such as SoilGrids is needed more than ever. For example, it is clear that in order to improve SoilGrids, more focus will need to be put on improving the feature space representation (adding extra samples) and on improving visualization and interpretation of complex relationships. Such improvements are not possible without understanding principles of spatial sampling and soil-environment relationships. Expert knowledge on soil-landscape relations and soil distribution remains important to evaluate the results and assess if predicted spatial patterns make sense from a pedological viewpoint. Even though the existing machine learning methods have proven to show improved predictive performance, much work remains to make them more robust, less sensitive to blunders, incorporate soil-landscape process knowledge and make them more suited for input data of variable accuracy.

With the current version of SoilGrids, we have also not yet adequately addressed the problem of vertical soil stratigraphy. At this stage, we remain unable to properly model how some soil horizons show smooth transition of soil properties, and some show clear and abrupt discontinuities (as in geological layers on a meso-scale). In the next update of SoilGrids we hope to improve modeling and prediction of occurrence of diagnostic soil horizons (e.g. Histic, Nitic, Albic etc) in 3D, so that transitions between horizons can be represented more accurately.

Preparation and conversion of soil class input data could also be much improved. Several research groups [67, 68] are now looking into automating soil classification (i.e. by using automated or semi-automated soil classification software). Eberhardt [69], for example, demonstrated using German soil profile data that soil classification can be completely automatized. Future versions of SoilGrids could also try to derive soil classes by applying exact rules per pixel, instead of trying to predict them from point data. This might be an ambitious project—often the classification systems (keys and rules of classification) can be very detailed and require a comprehensive combination of diagnostic properties, laboratory data, soil-moisture and temperature regimes, etc. in order to deduce the correct classification. This is without considering the sensitivity of such classifiers to data gaps and uncertainties. Incorporating uncertainty into such complex soil classification algorithms is yet another challenge. So far, we have managed to produce global maps of the scaled Shannon Entropy index (Fig 9) that clearly indicate under-represented areas. A sensible approach to improving predictions of soil types would be to set the sampling intensity proportional to the Shannon Entropy index or completely focus on areas where the Shannon Entropy index is > 80%. In that sense, there seems to be slightly more work needed for the WRB classification system than for the USDA system.

We have also so far explicitly avoided trying to model posterior distributions of target variables, i.e. map uncertainty for each soil variable. Although tools for modeling uncertainty in ML methods already exist (see e.g. Meinshausen [70]), these are hundreds of times more computationally intensive and will probably need to be re-implemented in some high-performance computing infrastructure. One future objective is to implement a framework to model uncertainties of all predictions using a robust statistical framework, such as quantile regression forests, but this might be highly challenging, especially when the data volumes grow larger.

Another opportunity for improvement lies in using spatiotemporal modeling [71, 72] vs purely spatial modeling. Stockmann et al. [2] recently made progress in modeling global soil organic carbon dynamics, mainly using time-series of MODIS land cover images, but numerous challenges remain:

- There might not be enough well-distributed soil profile data in the time-domain that support fitting of spatiotemporal (and/or dynamic) models. As we move back further in the past, there are fewer and fewer observations, so potential time-domain gaps are possibly an order of magnitude more serious than spatial data gaps,
- Some soil properties such as soil water content, soil temperature, and even soil nutrients, change not simply within seasons, but also within weeks or days. At this stage, global fitting of spatiotemporal models for such variables that vary at short time scales might remain unattainable (until new global soil monitoring networks are established),
- Legacy soil profile data exhibit a significant noise (diversity of methods, laboratories) so that, for example for soil organic carbon, where temporal dynamics are slow, it will be difficult to detect real changes in time in a situation where the signal-to-noise ratio is low,
- It is almost impossible to properly validate spatiotemporal predictions produced for past periods of time. There are very few and sparse validation soil data collected using objective probability sampling designs (as described in Brus et al. [60]). Eventually, we might never know how accurate our models are in predicting the past status of soil from 50 or 100 years ago. One possible solution to this problem is linking soil science more directly with paleontology and archeology, but this will probably not work for all soil variables.

### Predicting at resolutions finer than 250 meter

Because the algorithms and software we have used in this work are already optimized for processing large data, this opens a possibility to further speed up model fitting and prediction and to generate predictions at ever finer resolutions. Fig 11 identifies some new remote sensing data land products of relevance to global soil mapping. Note that some remote sensing products, such as Landsat 8 and ASTER (distributed as scenes), require significant processing capacities before they can be assembled and prepared for use in global soil mapping. Nevertheless, considering the amount of remote sensing data available publicly today, we anticipate that the Open Source software used in this work will soon (12–24 months) be able to support generation of 30 m resolution SoilGrids, provided that enough resources exist to cover the costs of preparing soil covariates and producing global predictions at these fine resolutions.

Presently, the biggest challenges for upgrading SoilGrids to finer resolution are the resources required to prepare all required remote sensing input data and computational capacity needed to make fine resolution predictions globally. The software seems to be much less of a problem. Although R has been often criticized for not being suited for large GIS layers, our experience with SoilGrids has convinced us that, with proper combination of parallelization and tiling of objects, and by using packages implemented in C++ or similar, equally efficient computing can be achieved with ranger and xgboost (hence within R) or by using software such as h2o (based on Java). The remaining bottleneck of R we experienced was the size of models produced using random forest—the objects often exceeded 5–10 GiB and as such require significant RAM during predictions. Such memory problems in R could possibly be solved via the following two strategies:

1. Disk caching: by using the ff or a similar packages to save the forests on disk,
2. Efficient tree representation: transform trees to a simpler structure with the same output.

In the case of random forest, the number of trees required for a given accuracy depends on the number of rows and columns, i.e. the number of observations (*n*) and covariates (*p*). Usually, for many rows only few trees are required, while for *p* ≫ *n* problems (for example in genetics) many more trees are needed. It should be generally fine to reduce the number of trees to fewer than 300 but this could be at the expense of loss in accuracy. Lopes [73] shows a framework, based on bootstrapping, to detect an optimal number of trees given some error threshold. For example, in many cases, even 150 trees is sufficient to achieve stable results after which a trade-off between computation time and accuracy offers no additional advantages. We have not tried fine-tuning the number of trees per property (we consistently use 300 trees as a practical compromise between precision and computing time) because this would have been an additional load to the project.

Another serious challenge to producing finer resolution SoilGrids is the current lack of adequately detailed geological data, i.e. data to represent the underlying lithology and mineralogy. We have thus far used the Global Lithological Map (GLiM) [35] as the key layer to represent parent material, but this layer is probably even coarser than 1 km resolution remote sensing covariates, and still contains numerous artifacts such as country/state borders. Although the OneGeology initiative is of obvious interest to global soil mapping projects, it has not, so far, delivered any globally consistent and complete information on parent material. Likewise, the latest most accurate DEM of the world (WorldDEM^™^) is an order of magnitude more accurate and more detailed than the SRTM DEM [74] and as such would be an ideal covariate for many regional and global soil mapping projects. However, it will likely remain a commercial product available to larger business only (civil engineering and mineral exploration), and hence of limited use to global soil mappers. In that sense, USA’s NASA and USGS, with its MODIS, Landsat and similar civil-applications missions will likely remain the main source of spatial covariate data to support global soil mapping initiatives.

Other potentially useful covariates for predicting soil properties and classes could be maps of paleolithic i.e. pre-historic climatic conditions of soil formation, e.g. glacial landscapes and processes, past climate conditions and similar. These could likely become significant predictors of many current soil characteristics. Information on pre-historic climatic conditions and land use is unfortunately often not available, especially not at detailed cartographic scales, although there are now several global products that represent, for example, dynamics of land use / changes of land cover (see e.g. HYDE data set by Klein et al. [75]) through the past 1500+ years. As the spatial detail and completeness of such pre-historic maps increases, they will become potentially interesting covariates for global soil modeling.

### Merging global and local: A system for automated soil image fusion

**Fig 12:**
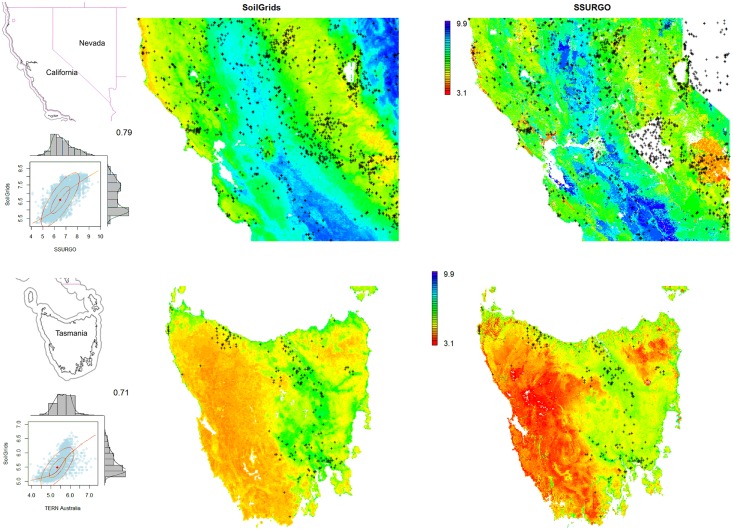
Comparison between predicted soil pH: (above) SoilGrids (our predictions) for part of California and predictions based on the SSURGO data set (for 0–200 cm depth interval) developed by the National Cooperative Soil Survey, (below) SoilGrids (our predictions) for Tasmania and predictions based on the Soil and Landscape Grid of Australia [76] (for 0–5 cm depth interval). The correlation coefficients between the two data sources are 0.79 and 0.71, respectively. Crosses on the map indicate soil profiles used for generating SoilGrids.

SoilGrids is not expected to be as accurate or relevant as locally produced maps and models that make use of considerably greater amounts of local point data and finer local covariates. This is especially the case for OECD countries that can draw upon orders of magnitude more soil profile data than were used in this work (for illustration, it is estimated that German Federal agencies alone have in possession 2–3 million complete soil profiles). Comparison of SoilGrids with similar national or continent-wide products shows that there is a general match in spatial patterns for many physical and chemical soil properties, although there are still substantial differences (Fig 12). This indicates that promising possibilities exist for further combination of local and global predictions (see further discussion).

For both Tasmania and California, SoilGrids seems to show somewhat smoother predictions, with some smoothing of higher and lower values, which is especially visible in the cross-histogram scatter plots (Fig 12). SoilGrids tends to overestimate soil pH for parts of Tasmania covered with rainforests mainly. There were not many ground observations to support the prediction models for those areas, hence some systematic deviation could be expected and will likely occur in other similar areas as well. We did not run a systematic comparison of values for all soil properties, but Fig 12 indicates that merging SoilGrids250m with 100m resolution predictions using higher density of local soil profiles could help to gradually improve accuracies locally and to fill gaps in locally generated predictions.

Mulder et al. [10] correctly recognized that, in many areas in the world, locally produced predictions of soil properties could likely be significantly more accurate than SoilGrids. Our hope is, nevertheless, that SoilGrids250m will be used by national and regional soil data production teams with, or as a supplement to, local data, and that ultimately most users will use merged (ensemble) global-local predictions for final decision making. We especially recommend the following two frameworks for combining global and local data:

1. SoilGrids predictions as covariate layers for producing finer resolution local predictions of soil properties (i.e. as an input for downscaling),
2. Ensemble predictions = SoilGrids + local soil spatial prediction models combined.

**Fig 13:**
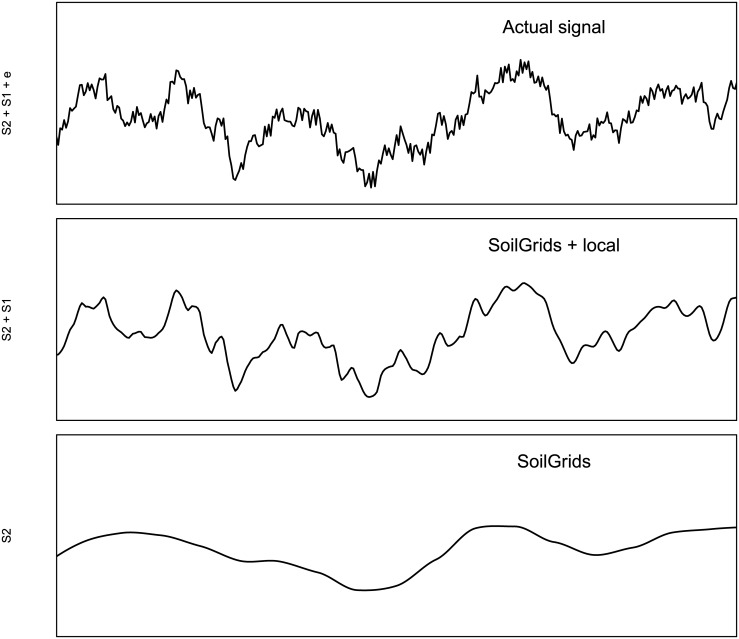
SoilGrids can be considered the ‘coarsest’ component of the global soil variation ‘signal’ curve. Other components, e.g. finer products based on local / more detailed 250–100 m resolution imagery, could be added to produce a merged product.

Option 2, i.e. produce ensemble predictions for smaller areas for which finer resolution and/or higher quality soil covariates are available, is possibly the most attractive option considering that local and global predictions can then be generated independently. In that sense, SoilGrids could also be considered to be just one (the coarsest) component of a global soil variation curve (Fig 13). But how many components to use to represent soil variation? Are two components enough? How to optimally merge components where the accuracy is unknown (not enough ground data for validation)? These will be areas of further research. In that context, Malone et al. [77] recently made progress in testing and developing methods for merging predictions from polygon-based maps and maps derived using spatial predictions. However, running such models in an automated way for large areas (i.e. a system for an automated soil image fusion) might take years before an operational system for global soil data fusion is fully functional.

## Conclusions

Soil has long been considered one of the least developed global environmental layers with data available only at coarse resolutions and with limited accuracy [78, 79]. ISRIC—World Soil Information has a vision and a mission to produce soil information and map products that are globally complete and consistent, scientifically robust, open, transparent and reproducible, continuously improved and updated, easy to discover and access, easy to use and meaningful to users. With this next generation SoilGrids250m we hope to continue to demonstrate progress in the production and distribution of improved global soil map products and to motivate, especially non-soil scientists, to use these new soil data in their models and spatial planning, i.e. directly as input for generation of soil functional properties and agro-ecological variables and indicators to support decision making. With its Open Data license and web-services, we aim to serve quality soil information freely and universally for science, society and a sustainable future.

**Fig 14:**
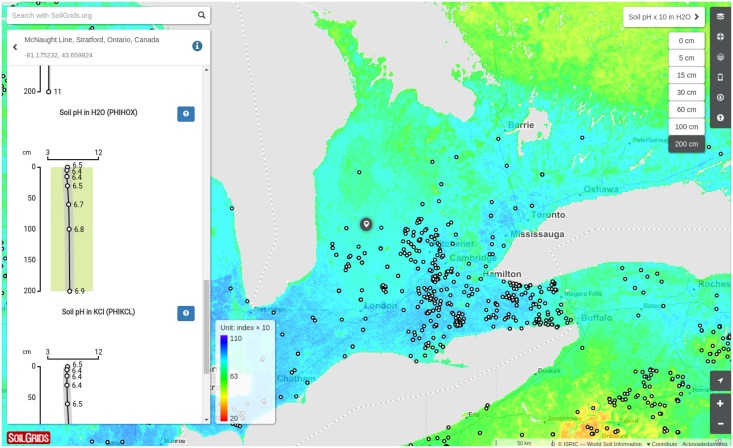
Basic design and functionality of SoilGrids.org: Soil web-mapping browser that provides interactive viewing of 3D soil layers. Reference administrative data, basic functionality and output data license of SoilGrids.org are primarily based on OpenStreetMap.

We have demonstrated, using a series of cross-validation tests, that the new version of SoilGrids represents a significant improvement upon the previous products at 1 km resolution, especially in terms of spatial detail and attribute accuracy. Future work is required to determine if these improvements in accuracy could also help produce more accurate Global Gridded Crop Models (GGCMs) that allow for more reliable estimates of impact of climate change and land degradation on food production [8]. Data accessibility problems with SoilGrids have also been addressed: SoilGrids are now available for viewing in fusion with satellite imagery via the data portal SoilGrids.org (Fig 14). SoilGrids rasters can also be downloaded via FTP for smaller areas; at point locations through the SoilInfo App and the REST SoilGrids. There should be fewer and fewer obstacles for ecologists, agronomists, hydrologists, climatologists, foresters and spatial planners to discover, obtain and use soil data in their daily work.
